# Linking between soil properties, bacterial communities, enzyme activities, and soil organic carbon mineralization under ecological restoration in an alpine degraded grassland

**DOI:** 10.3389/fmicb.2023.1131836

**Published:** 2023-04-06

**Authors:** Xiangyang Shu, Yufu Hu, Weijia Liu, Longlong Xia, Yanyan Zhang, Wei Zhou, Wanling Liu, Yulin Zhang

**Affiliations:** ^1^College of Resources, Sichuan Agricultural University, Chengdu, China; ^2^Chengdu Academy of Agriculture and Forestry Sciences, Chengdu, China; ^3^Institute for Meteorology and Climate Research (IMK-IFU), Karlsruhe Institute of Technology, Karlsruhe, Baden-Wurttemberg, Germany; ^4^Department of Civil Engineering, The University of Hong Kong, Pokfulam, Hong Kong SAR, China

**Keywords:** ecological restoration, enzyme activity, soil microorganisms, carbon mineralization and storage, alpine grassland

## Abstract

Soil organic carbon (SOC) mineralization is affected by ecological restoration and plays an important role in the soil C cycle. However, the mechanism of ecological restoration on SOC mineralization remains unclear. Here, we collected soils from the degraded grassland that have undergone 14 years of ecological restoration by planting shrubs with *Salix cupularis* alone (SA) and, planting shrubs with *Salix cupularis* plus planting mixed grasses (SG), with the extremely degraded grassland underwent natural restoration as control (CK). We aimed to investigate the effect of ecological restoration on SOC mineralization at different soil depths, and to address the relative importance of biotic and abiotic drivers of SOC mineralization. Our results documented the statistically significant impacts of restoration mode and its interaction with soil depth on SOC mineralization. Compared with CK, the SA and SG increased the cumulative SOC mineralization but decreased C mineralization efficiency at the 0–20 and 20–40 cm soil depths. Random Forest analyses showed that soil depth, microbial biomass C (MBC), hot-water extractable organic C (HWEOC), and bacterial community composition were important indicators that predicted SOC mineralization. Structural equal modeling indicated that MBC, SOC, and C-cycling enzymes had positive effects on SOC mineralization. Bacterial community composition regulated SOC mineralization *via* controlling microbial biomass production and C-cycling enzyme activities. Overall, our study provides insights into soil biotic and abiotic factors in association with SOC mineralization, and contributes to understanding the effect and mechanism of ecological restoration on SOC mineralization in a degraded grassland in an alpine region.

## Introduction

The alpine grasslands on the Qinghai-Tibetan Plateau, which cover roughly 40% of China’s grassland area, serve as an essential ecological barrier and carbon sink (Chen et al., 2022; Wang Y. et al., 2022). However, due to human disturbances and climate changes, degradation of alpine grasslands is widespread and has accelerated in the past decades, resulting in a significant loss of biodiversity and soil C stocks (Bardgett et al., 2021). Ecological restoration is one of several actions that can ameliorate degraded and disturbed soils, with the goal of rebuilding, initiating, or accelerating recovery of disturbed ecosystems (Martin, 2017). Restoration activities can reverse soil degradation, mitigate climate change, and combat the loss of biodiversity and ecosystem services (Dong et al., 2020). It is known that the effect of ecological restoration on the soil C pool depends on the balance between C input from plants and C effluxes *via* microbial mineralization (Jackson et al., 2017; Dynarski et al., 2020). Meanwhile, carbon dioxide (CO_2_) mitigation and soil fertility maintenance can both be achieved through reducing the process of SOC mineralization (Zhang B. et al., 2021; Zhang S. et al., 2021; Dong et al., 2022). By slowing down the rate of SOC mineralization and increasing SOC content, it is possible to reduce the release of CO_2_ into the atmosphere and maintain soil health. To date, our understanding of how ecological restoration affects SOC mineralization and its mechanism in alpine grasslands lags considerably behind that of SOC storage (Zhou et al., 2022). These knowledge gaps undermine our predictions of ecological restoration effects on soil C processes and constrain the improvement of restoration management practices to resist land degradation.

Soil physiochemical properties are essential factors affecting SOC mineralization (Zhao et al., 2008; Ahn et al., 2009). For instance, soil pH affects SOC mineralization by altering microbial communities and enzyme activities (Zhang et al., 2022; Zhuang et al., 2022). Soil nutrient availability, such as nitrogen and phosphorus, can also impact SOC mineralization by impacting microbial activities (Jing et al., 2017; Wei et al., 2020; Peixoto et al., 2021). For instance, when nitrogen or phosphorus is limiting, microbes may switch from using organic carbon to using other sources of carbon, reducing the rate of SOC mineralization. Meanwhile, the labile C fractions, such as microbial biomass carbon (MBC), easily oxidized carbon (EOC), and hot-water extractable carbon (HWEOC), serve as the main C sources for microorganisms that determine SOC mineralization (Rousk et al., 2016). Therefore, understanding the variation in soil physiochemical properties and carbon fractions and their relations to SOC mineralization under ecological restoration could improve our ability to make accurate predictions.

Soil microbiota constitute a large part of the earth’s biodiversity and are involved in C sequestration, SOM decomposition, and nutrient cycling and availability (Liang et al., 2017; Banerjee et al., 2018; Yang et al., 2018; Crowther et al., 2019; Shu et al., 2022). Therefore, any changes in the diversity, composition, and potential functions of microbial communities may alter the direction and magnitude of SOC mineralization (Schimel and Schaeffer, 2012; Juarez et al., 2013; Tardy et al., 2015; Zhang et al., 2019; Ibrahim et al., 2021). Microbial extracellular enzymes, especially C-cycling enzymes (e.g., *β*-1,4-glucosidase, *β*-d-cellobiosidase, peroxidase, polyphenol oxidase), play an essential role in the decomposition of SOC and the regulation of C fractions (Chen et al., 2018a; Yang et al., 2019; Chen J. et al., 2020). Ecological restoration may affect microbial community structure and enzyme activities through a direct effect of *via* regulating the quantity and quality of litter inputs, and through an indirect effect of modifying soil physiochemical properties (Deng et al., 2010; Raiesi and Salek-Gilani, 2018; Xu et al., 2021; Yang et al., 2022). Therefore, soils under different ecological restoration modes may differ in microbial community structure and enzyme activities and consequently the SOC mineralization. However, limited data are available regarding the comprehensive influences of soil physiochemical properties, microbial community composition, and enzyme activities on SOC mineralization.

Here, we explored how ecological restoration may influence SOC mineralization and its relation to soil physiochemical properties, labile carbon fractions, enzyme activities and bacterial communities in degraded grasslands on the Tibetan Plateau. The primary aims of this study were: (1) to explore changes in soil physiochemical properties, labile carbon fractions, bacterial communities, and enzyme activities after 14-year restoration treatments; (2) to determine the influence of ecological restoration on SOC mineralization; and (3) to identify the relative importance of biotic and abiotic factors in determining SOC mineralization under ecological restoration.

## Materials and methods

### Site description

The study area is located in the Restoration Demonstration region of a degraded grassland in Hongyuan County (33°1’ N and 102°37′ E), China, at the eastern margin of the Tibetan Plateau (Supplementary Figure S1). The average elevation of this region is over 3,400 m. The mean annual precipitation in this region is 791.95 mm. The mean annual temperature is 1.1°C, and the mean temperatures are −10.3 and 10.9°C for the coldest and warmest months, respectively. The soil is classified as cambic arenosol (FAO Classification, 2006). The dominant vegetation species in the recovery area are mainly *Salix cupularis*, *Carex peaeclara*, *Kobresia pygmaea*, *Artemisia wellbyi*, and *Heracleum souliei*. Since 2007, the extremely degraded grassland at this site has undergone natural restoration with the dominant species being *Cyperus stoloniferus*; this was the control (CK) for the study. Two artificial restoration actions were started as well to restore the degraded grassland. The artificial restoration actions included: (1) planting shrubs with *Salix cupularis* alone (SA), and (2) planting shrubs with *Salix cupularis* plus mixed grasses (SG). The primary species in SA were *Salix cupularis*, *Lancea tibetica*, and *Leymus secalinus*. The primary species in SG were *Salix cupularis*, *Euphrasia regelii subsp. Kangtienensis*, *Anaphalis lacteal*, *Peucedanum praeruptorum*, *Potentilla discolor*, and *Elymus nutans*. At the time of our study, the natural and artificial restoration actions had been ongoing for 14 years.

### Experimental design

In August 2021, soil samples were taken from three areas in an extremely degraded grassland; one that underwent natural restoration (CK), one that was planted with shrubs and *Salix cupularis* alone (SA), and one that was planted with shrubs and *Salix cupularis* plus grasses (SG). Four independent plots were selected for each treatment, where four quadrats, each 1 m × 1 m were set up. The characteristics of the vegetation community were examined in the field before collecting soil samples (Supplementary Table S1). We randomly sampled 1 kg of soil from the 0–20 cm and 20–40 cm soil layers in each plot using a 5-cm diameter soil auger. Then, we pooled and thoroughly mixed the samples to produce a composite soil sample. In total, 24 samples (3 treatments × 4 replicates × 2 depths) were collected. After transporting these samples to the laboratory on ice, one-tenth of each soil sample was stored at −80°C for the soil bacterial community analysis. Two-tenths of each soil sample was stored at 4°C for testing soil microbial biomass carbon and enzyme activities. The remaining soil was air-dried and sieved for pH, soil organic carbon (SOC), and soil nutrients analysis. Moreover, a cutting ring with a capacity of 100 cm^3^ was used to collect undisturbed soil before performing soil bulk density analysis.

### Soil physicochemical characterization

Soil physicochemical characteristics were analyzed as previously described by Carter and Gregorich (2007). Soil pH was determined by a glass electrode with a soil-to-water ratio of 1:2.5 (weight/volume) (Mettler Toledo MP220, Mettler-Toledo, Switzerland). SOC content was analyzed using the K_2_Cr_2_O_7_ oxidation method. Soil total nitrogen (TN) content was measured using a flow injection autoanalyzer (AutoAnalyzer 3, Bran+ Luebbe, Germany). Soil total phosphorus (TP) content was analyzed calorimetrically using the H_2_SO_4_-HClO_4_ method. Bulk density was examined by the cutting ring method, undisturbed soil samples were dried at 105°C to reach a constant weight.

### Soil labile carbon fractions and C-cycling enzymes

Microbial biomass carbon (MBC) was measured by the chloroform fumigation-extraction method (Vance et al., 1987). Hot-water extractable organic carbon (HWEOC) was determined using a TOC analyzer (Elementer Analysensysteme, Germany) (Hou et al., 2021). Easily oxidized carbon (EOC) was measured according to the 333 mol L^−1^ KMnO_4_ method as described by Dong et al. (2022). Additionally, we analyzed the potential activities of four C-cycling enzymes, including β-glucosidase (BG), β-d-cellubiosidase (CBH), peroxidase (POD), and polyphenol oxidase (PPO). All enzymes were measured using commercial enzyme kits following the manufacturer’s protocol (Solarbio Science and Technology Co., Ltd., Beijing, China).

### Soil C mineralization

Cumulative SOC mineralization was determined according to the method described by Hou et al. (2021). First, two 25 ml glass beakers filled with 10 g fresh soil and 15 ml 1 M NaOH solution, respectively, were put side by side in an airtight plastic 250 ml jar. Deionized water was spread on the bottom of the jar and surrounded the breakers to keep constant soil moisture. Then, these 250 ml jars were placed in a thermostatic incubator at 25°C for 28 days. During the incubation, the CO_2_ gas generated was absorbed in the NaOH solution, and the remaining NaOH was measured by titrating with 0.1 M HCl to quantify SOC mineralization.

### DNA extraction and Illumina MiSeq sequencing

For each sample, total DNA was extracted from 0.5 g soil using the PowerSoil® DNA Isolation Kit (MoBio Laboratories Inc., Carlsbad, CA, United States) following the manufacturer’s instructions. The concentration and quality of DNA were measured by Nanodrop 2000 (Thermo Scientific, Wilmington, DE, United States). Before performing PCR amplification, the DNA sample was diluted to 10 ng/μL. The 16S rRNA V4–V5 regions were sequenced for bacterial communities with the primer pair 515F (5′-GTGCCAGCMG CCGCGGTAA-3′) and 909R (5′-CCCCGYCAATTCMTTTRAGT-3′). Sequencing was conducted on an Illumina MiSeq2500 platform by Novogene (Beijing, China). The raw sequence data of the 16S rRNA were analyzed using the Quantitative Insights into Microbial Ecology (QIIME) pipeline. Using a dissimilarity level of 3%, the unique sequence set clustered operational taxonomic units (OTUs) into the UPARSE pipeline.

### Functional analysis of the bacterial community using PICRUSt2

Changes in functional genes involved in C cycling (including C degradation and C fixation) were predicted by phylogenetic investigation of bacterial communities by reconstruction of unobserved states 2 (PICRUSt2) according to the Kyoto Encyclopedia of Genes and Genomes (KEGG) database and 16S rRNA bacterial community data (Li et al., 2022). The KEGG orthologues of each gene generated by PICRUSt2 were obtained from the table of absolute abundance for the KEGG pathway, which then was converted into the relative abundance of the corresponding genes.

### Calculation of indices

Stocks of SOC were calculated using Equation 1 (Hu et al., 2018):


(1) $$\begin{array}{l} \mathrm{SOC}\;\mathrm{stock}\;\left(\mathrm{Mg}\;{\mathrm{ha}}^{-1}\right)=\mathrm{SOC}\;\left(g\;{\mathrm{kg}}^{-1}\right)\times \mathrm{bulk density}\;\\ \;\;\;\;\;\;\;\;\;\;\;\;\;\;\;\;\;\;\;\;\;\;\;\;\;\;\;\;\;\;\;\;\;\;\;\;\;\;\;\;\;\;\;\;\;\;\;\;\;\;\;\;\;\;\;\;\left(g\;{\mathrm{cm}}^{-3}\right)\times \mathrm{soil depth}\;\left(\mathrm{cm}\right)/10 \end{array}$$

where SOC stock indicates the soil organic carbon stock, and SOC indicates the soil organic carbon content.

The following Equation 2 was adopted to calculate carbon mineralization efficiency (Dong et al., 2022):


(2) $$\begin{array}{l} \mathrm{CME}\;\left(\mathrm{mg}\;{\mathrm{CO}}_{2}-C\;g^{-1}\mathrm{SOC}\right)=\mathrm{Cumulative}\;\mathrm{SOC}\;\;\\ \;\;\;\;\;\;\;\;\;\;\;\;\;\;\;\mathrm{mineralization}\;\;\left(\mathrm{mg}\;{\mathrm{CO}}_{2}-C\;{\mathrm{kg}}^{-1}\right)/\mathrm{SOC}\;\left(g\;{\mathrm{kg}}^{-1}\right) \end{array}$$

where CME indicates the carbon mineralization efficiency and SOC indicates the soil organic carbon content.

### Statistical analyses

Statistical analyses were conducted using the R statistical software (R version 4.0.2, R Core Team, Vienna, Austria). Unless otherwise stated, statistical significance was set at *p* < 0.05. Difference in soil physiochemical properties, labile C fractions, enzyme activity, and SOC mineralization between different treatments at two different soil depths were tested using a two-way analysis of variance (ANOVA). When two-way ANOVA revealed differences, a Tukey’s honestly significant difference (Tukey HSD) test was used to compare the average value of variables among the different treatments. Linear regression analysis was used to evaluate the relationships between soil physiochemical properties, labile C fractions, the diversity and composition of bacterial communities, enzyme activities and SOC mineralization. Principal coordinates analysis (PCoA) was used to determine significant differences in microbial communities for the various restoration modes and soil depths. Redundancy analysis (RDA) was performed with a Monte Carlo permutation test (999 permutation) to identify soil properties that influence the bacterial community structure. The Mantel test was performed to identify soil variables that influence the microbial community structure. We performed random forest analysis to evaluate important predictors of SOC mineralization among soil depth, physiochemical properties, labile C fractions, enzyme activities, bacterial Shannon index, bacterial Chao1 index, and bacterial composition. Bacterial community composition was estimated based on Bray–Curtis distances between samples. Random forest analysis was performed using the “randomForest” package, with the significance of the model and each predictor was evaluated using the “rfPermute” packages. Furthermore, we constructed structural equation modeling (SEM) to evaluate the direct and indirect effect of various variables on SOC mineralization under ecological restoration. Bacterial composition was represented by scaling 1, the first component of principal coordinates analysis. The goodness of fit of the SEM was evaluated using the Chi-square test, the whole-model *p* value, Akaike information criterion (AIC), and the goodness-of -fit (GFI) statistic. The SEM was conducted using AMOS software (IBM SPSS Amos 24.0.0).

## Results

### Soil physiochemical properties and labile C fractions

**Figure 1:**
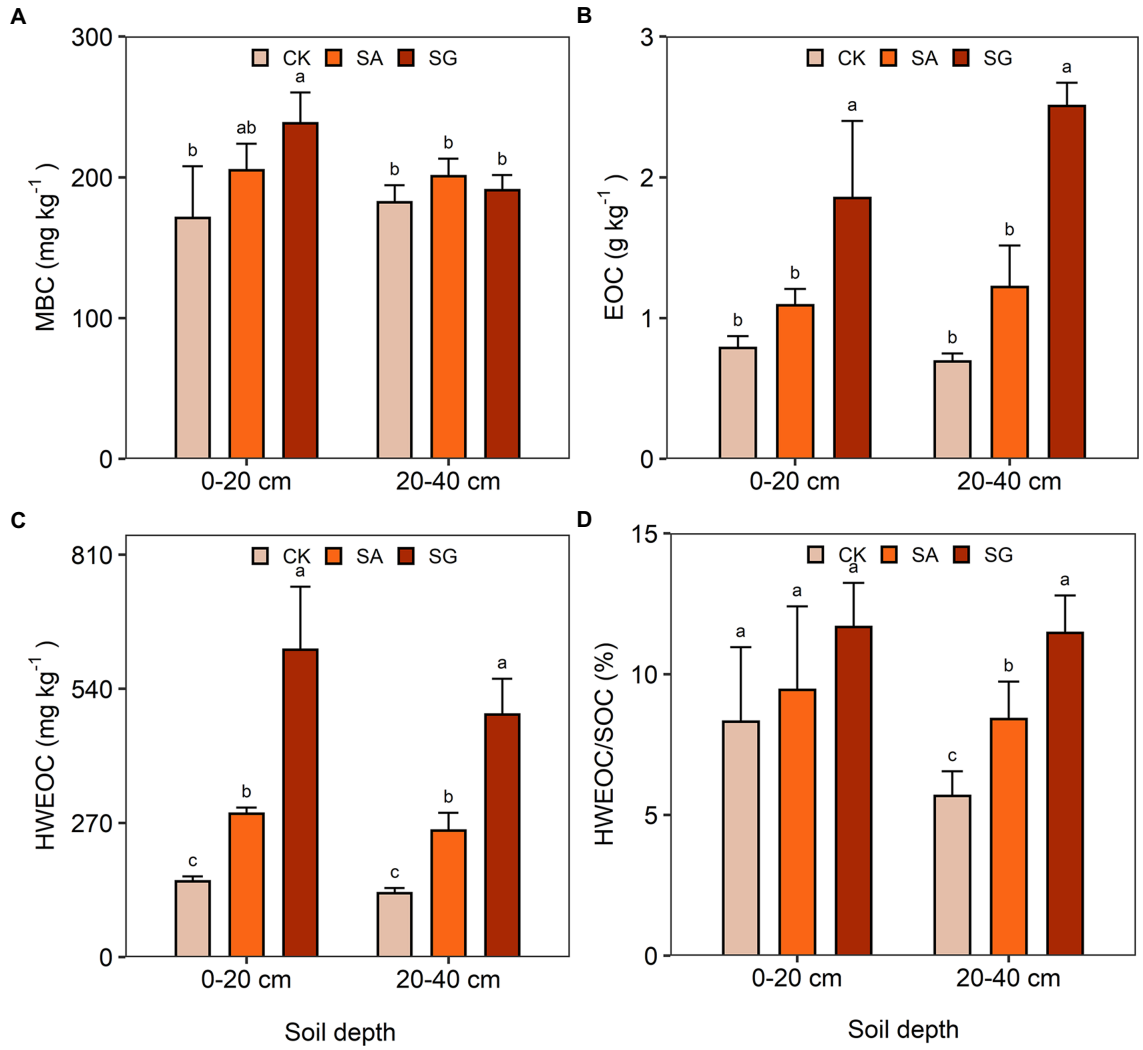
Effects of different restoration modes on soil labile carbon fractions. **(A)** Microbial biomass carbon (MBC), **(B)** easily oxidized carbon (EOC), **(C)** hot-water extractable organic carbon (HWEOC), **(D)** the ratio of hot-water extractable organic carbon to soil organic carbon (HWEOC/SOC). Error bars indicate standard deviation; Different lowercase letters indicate significant differences at *p* < 0.05 among treatments, based on the Tukey’s honest significance difference (HSD) test.

The two-way ANOVA demonstrated that soil pH significantly differed in restoration mode (*F* = 67.58, *p* < 0.001), soil depth (*F* = 6.36, *p* < 0.05), and by the interaction of restoration mode and soil depth (*F* = 4.00, *p* < 0.05) (Supplementary Table S2). Compared with CK, SA significantly decreased soil pH at the 0–20 and 20–40 cm soil depths (*p* < 0.05). Restoration mode had a significant effect on SOC (*F* = 18.07, *p* < 0.001), TN (*F* = 91.85, *p* < 0.001), and SOC stock (*F* = 17.97, *p* < 0.001), but had no significant effect on soil BD and TP. On average, the SOC content, TN content and SOC stock followed the order of SG > SA > CK (Table 1). Restoration mode had a significant effect on MBC (*F* = 6.94, *p* < 0.01), EOC (*F* = 60.20, *p* < 0.001), HWEOC (*F* = 93.34, *p* < 0.001), and HWEOC/SOC (*F* = 8.43, *p* < 0.001). Soil depth had a significant effect on HWOEC (*F* = 6.20, *p* < 0.05). Moreover, MBC (*F* = 4.26, *p* < 0.05) and EOC (*F* = 4.05, *p* < 0.05) significantly varied with the interaction of restoration mode and soil depth (Supplementary Table S2 and Figure 1).

**Table 1:** Effects of different restoration modes on soil physiochemical properties in different soil depths.

Soil depth	Variable	CK	SA	SG
0–20 cm	pH	6.81 ± 0.08 a	6.27 ± 0.18 b	6.68 ± 0.04 a
	BD (g cm^−3^)	1.42 ± 0.04 a	1.35 ± 0.05 a	1.41 ± 0.07 a
	TN (g kg^−1^)	0.11 ± 0.02 b	0.17 ± 0.02 b	0.39 ± 0.08 a
	TP (g kg^−1^)	0.16 ± 0.00 a	0.17 ± 0.01 a	0.18 ± 0.01 a
	SOC (g kg^−1^)	2.05 ± 0.78 b	3.39 ± 1.26 ab	5.39 ± 1.33 a
	SOC stock (Mg ha^−1^)	5.82 ± 2.29 b	9.15 ± 3.56 ab	15.01 ± 2.91 a
20–40 cm	pH	6.85 ± 0.03 a	5.95 ± 0.18 c	6.57 ± 0.15 b
	BD (g cm^−3^)	1.39 ± 0.13 a	1.38 ± 0.06 a	1.39 ± 0.09 a
	TN (g kg^−1^)	0.12 ± 0.02 b	0.18 ± 0.05 b	0.36 ± 0.03 a
	TP (g kg^−1^)	0.17 ± 0.01 a	0.17 ± 0.00 a	0.17 ± 0.00 a
	SOC (g kg^−1^)	2.30 ± 0.35 a	3.05 ± 0.27 a	4.31 ± 0.82 a
	SOC stock (Mg ha^−1^)	6.46 ± 1.38 a	8.40 ± 0.87 a	12.03 ± 2.88 a

Values are represented as the mean followed by a standard deviation in parentheses (*n* = 3). Different lowercase letters indicate a significant different (*p* < 0.05) among different modes, based on the analysis of variance, and Tukey’s honest significance difference (HSD) test. BD, bulk density, SOC, soil organic carbon; TN, total nitrogen; TP, total phosphorus; SOC stock, soil organic carbon stock.

### Bacterial community diversity and composition

**Figure 2:**
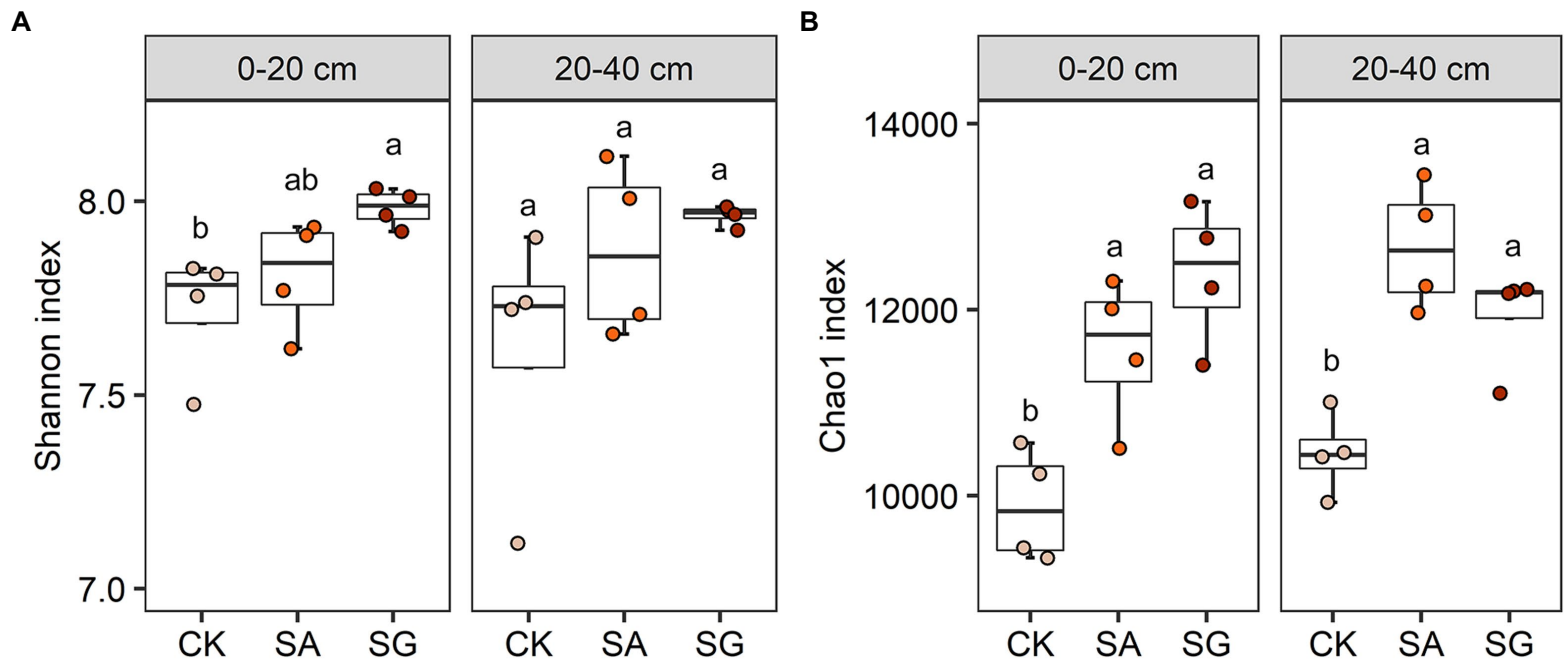
Effects of different restoration modes on bacterial alpha diversity. **(A)** Bacterial Shannon index and **(B)** bacterial Chao1 index. Error bars indicate standard deviation; different lowercase letters indicate significant differences at *p* < 0.05 among treatments, based on the Tukey’s honest significance difference (HSD) test.

Restoration mode had a significant effect on the Shannon index for bacteria in 0–20 cm soil layer (*p* < 0.05). The highest average value of the Shannon index at the 0–20 cm and 20–40 cm soil depths were observed in SG (Figure 2A). The Chao1 index for bacteria varied significantly with restoration mode (*p* < 0.05). Compared with CK, modes SA and SG significantly increased theChao1 index in the 0–20 cm and 20–40 cm soil depths (*p* < 0.05) (Figure 2B). SOC, TN, HWEOC, and EOC were positively correlated with the Chao1 and Shannon indices (*p* < 0.05). MBC was positively correlated with the Chao1 index (*p* < 0.05) (Supplementary Figure S2).

**Figure 3:**
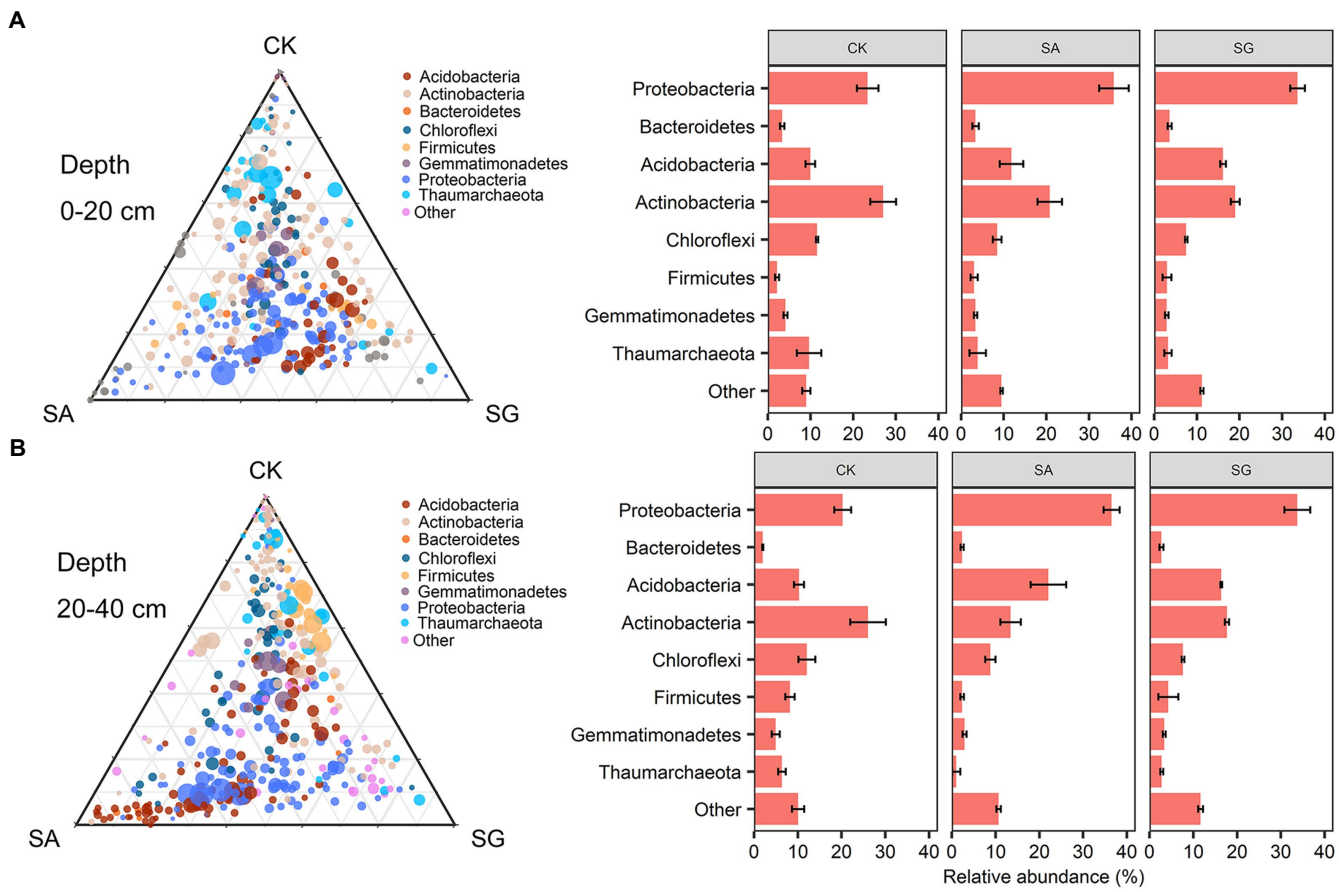
Taxonomic distribution of bacterial taxa responsible for community different among different restoration modes at 0–20 cm **(A)** and 20–40 cm **(B)** soil depth.

**Figure 4:**
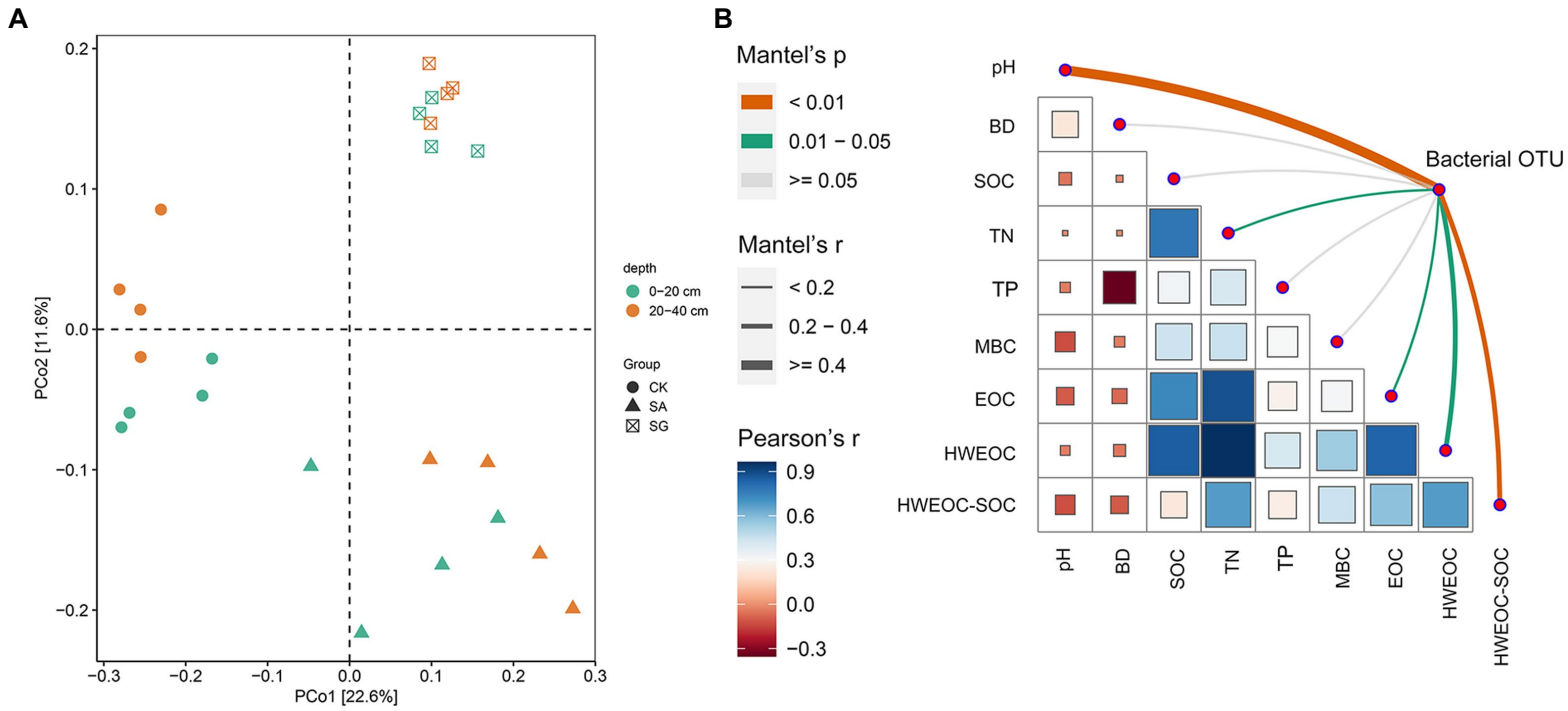
**(A)** Principial coordinates analysis (PCoA) of bacterial community composition based on Bray–Curtis distances. **(B)** Mantel test analysis of bacterial community changes with soil properties.

**Figure 5:**
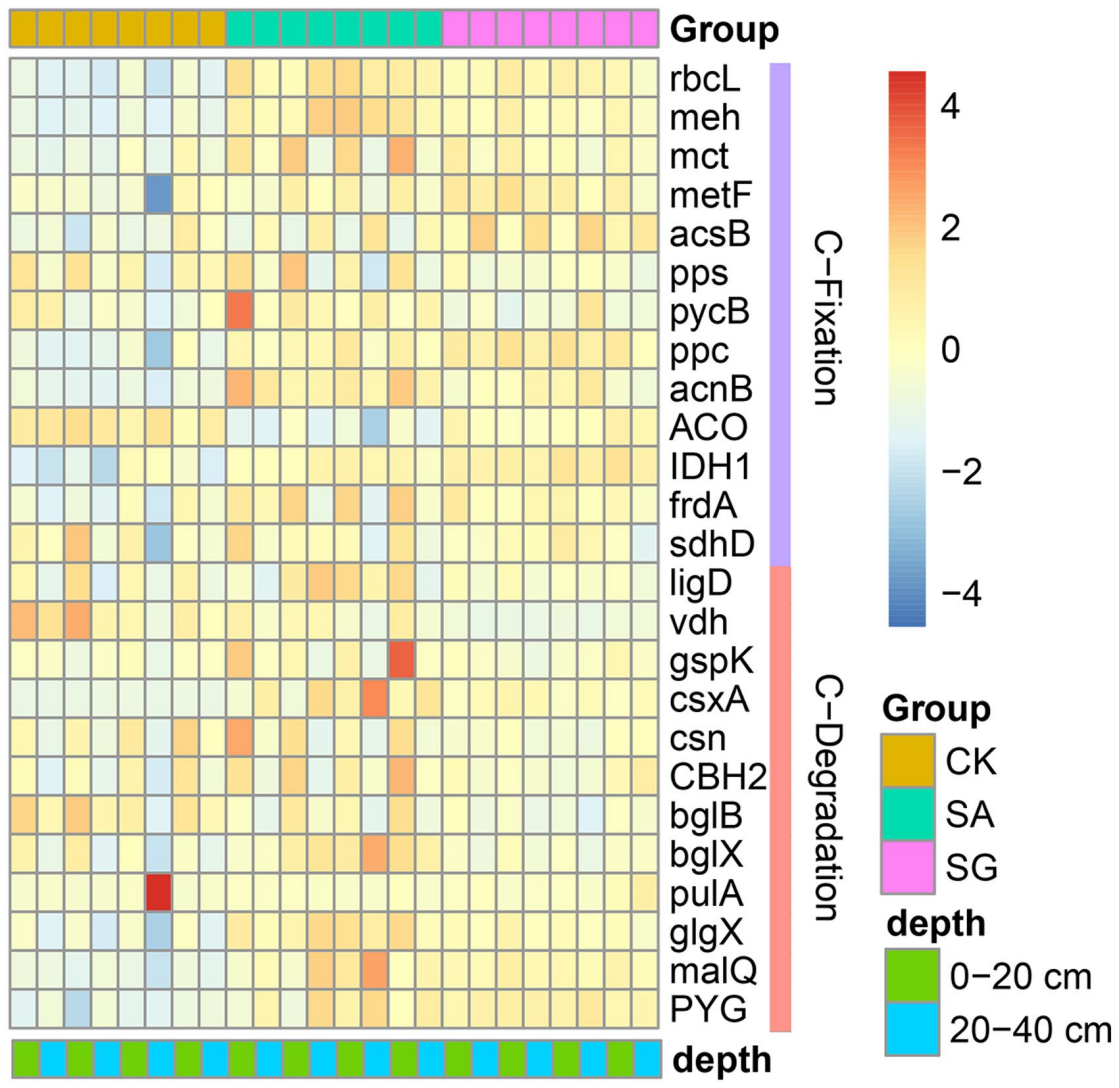
Effects of ecological restoration on the relative abundance of functional genes involved in C-cycling according to PICRUST2.

The most abundant bacterial phyla were *Proteobacteria*, *Actinobacteria*, *Acidobacteria*, and *Chloroflexi*. Compared with CK, modes SA and SG increased the relative abundance of *Proteobacteria*, *Acidobacteria*, and *Bacteroidetes*, but decreased the relative abundance of *Actinobacteria*, *Chloroflexi*, and *Thaumarchaeota* at the 0–20 cm and 20–40 cm soil depths (Figure 3). The PCoA analyses showed that the soil bacterial community in CK was separated from the soil bacterial community of soils in SA and SG (Figure 4A). RDA was used to identify the major soil properties controlling the soil bacterial community structure. The first two components explained 49.5% of the total variability for bacterial community structure. Soil pH, TN, EOC, and HWEOC were the important soil properties controlling the bacterial community structure (Supplementary Figure S3). The Mantel test indicated that soil pH, TN, EOC, HWEOC, and the ratio of HWEOC to SOC were the critical soil parameters affecting the bacterial community composition (Figure 4B). The PICRUST2 analysis indicated that ecological restoration significantly improved the role of microbes in C-fixation and decomposition. The relative abundance of C-fixation genes (*rbcL*, *meh*, *mct*, *ppc*, *IDH1*, and *frdA*) and C-degradation genes (*csxA*, *glgX*, *malQ*, and *PYG*) were higher in SA and SG than in CK (Figure 5).

### C-cycling enzyme activities

**Figure 6:**
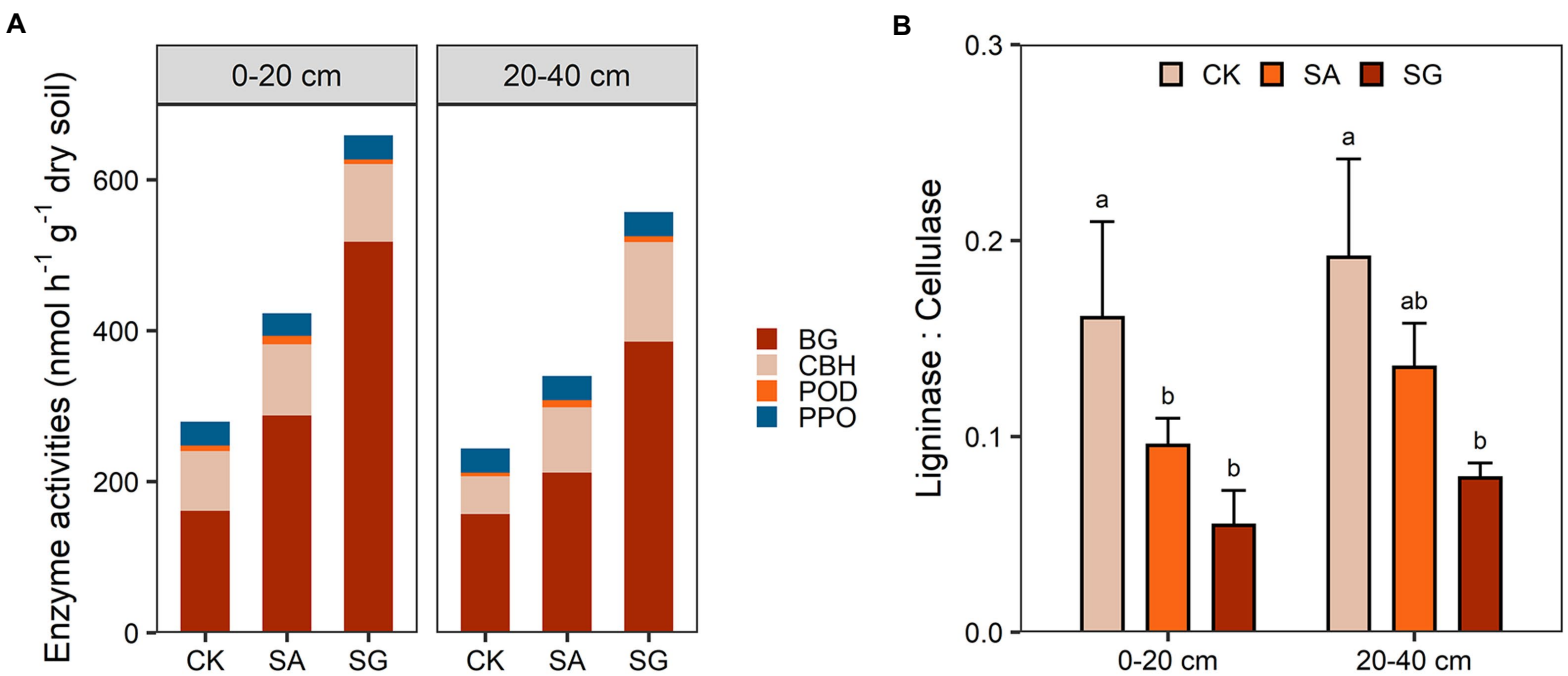
Effects of different restoration modes on soil carbon-cycling enzyme activities **(A)** and the ratio of ligninase to cellulase **(B)**. CK, extremely degraded grassland. SA, planting shrub with *Salix cupularis* alone (SA). SG, planting shrub with *Salix cupularis* plus mixed grasses. Error bars indicate standard deviation; different lowercase letters indicate significant differences at *p* < 0.05 among treatments, based on the Tukey’s honest significance difference (HSD) test.

Restoration mode had a significant effect on the activities of BG (*F* = 10.37, *p* < 0.01) and CBH (*F* = 4.88, *p* < 0.05), but had no significant effect on the activities of PPO and POD (Supplementary Table S4). Compared with CK, modes SA and SG increased the activities of BG and CBH in the 0–20 and 20–40 cm soil depths (Figure 6A). Modes SA and SG decreased the ratio of ligninase to cellulase by 40.6 and 66.0% in the 0–20 cm soil depth, and by 29.3 and 58.9% in 20–40 cm soil depth, respectively, relative to CK (Figure 6B). Both BG and CBH were positively correlated with TN, MBC, and HWEOC (*p* < 0.05) (Supplementary Figure S4). The ratio of ligninase to cellulase was negatively associated with SOC content and SOC stocks (*p* < 0.05) (Supplementary Figure S5).

### SOC mineralization

**Figure 7:**
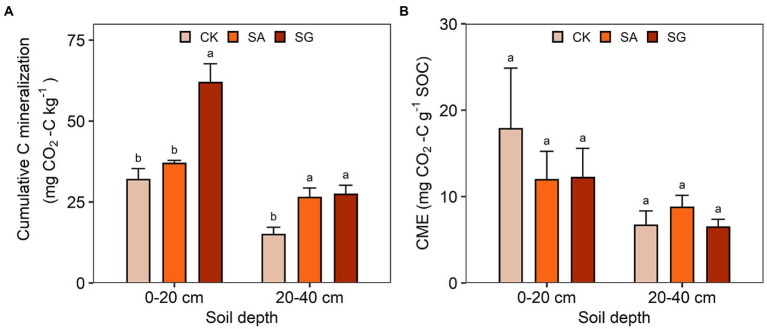
Effects of ecological restoration on **(A)** the cumulative carbon mineralization and **(B)** carbon mineralization efficiency (CME). Error bars indicate standard deviation; different lowercase letters indicate significant differences at *p* < 0.05 among treatments, based on the Tukey’s honest significance difference (HSD) test.

Restoration mode had a significant effect on cumulative C mineralization (*F* = 59.22, *p* < 0.001) (Supplementary Table S5). Compared with CK, the cumulative C mineralization at the 0–20 and 20–40 cm soil depth increased by 15.7 and 76.8% in the SA mode, and by 94.0 and 83.1% in the SG mode, respectively. The CME in the SG mode was lower than in CK (Figure 7). Soil depth had a significant effect on cumulative C mineralization (*F* = 166.07, *p* < 0.001) and CME (*F* = 15.78, *p* < 0.001). The cumulative C mineralization significantly varied with the interaction of restoration mode and soil depth (*F* = 20.06, *p* < 0.001) (Supplementary Table S5).

### Contribution of abiotic and biotic factors to SOC mineralization

**Figure 8:**
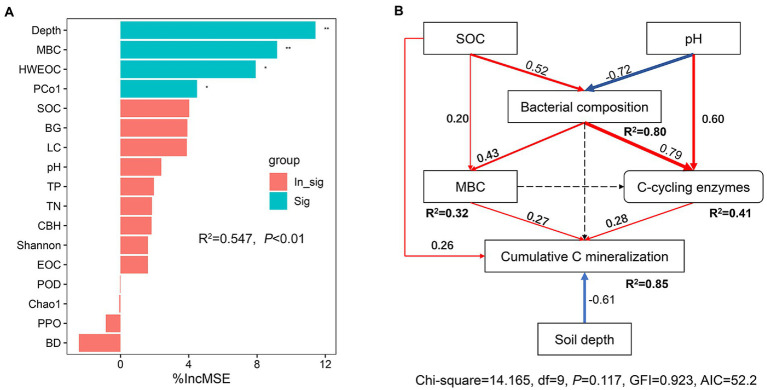
**(A)** Random Forest regression model shows the main factors of SOC mineralization. MSE, is the mean square error. ^*^p < 0.05, ^**^p < 0.01 on the bar indicated that the associated factor had a significant effect on SOC mineralization. **(B)** Structural equal model (SEM) analysis of the direct and indirect effects on the response of SOC mineralization to ecological restoration. Red and blue solid arrows indicate positive and negative relationships, respectively. Black arrows represent tested, but not significant paths. The arrow width is proportional to the strength of the relationship. Goodness-of-fit statistics for the model are shown below the model. ^*^p < 0.05, ^**^p < 0.01.

Random forest modeling indicated that the top six most important factors were soil depth, MBC, HWEOC, bacterial composition, and SOC (Figure 8A). SEM analysis showed that both SOC, MBC and C-cycling enzymes had a positive effect on the cumulative C mineralization, but soil depth had a negative effect on cumulative C mineralization (*p* < 0.05). Soil pH and SOC had a significant effect on bacterial composition (*p* < 0.05). Bacterial community composition had a significant positive effect on MBC and C-cycling enzymes (*p* < 0.05) (Figure 8B).

## Discussion

### Effects of ecological restoration on soil physiochemical characteristics and C fractions

Ecological restoration plays a critical role in maintaining soil quality *via* increasing nutrient contents, improving soil physical properties (e.g., aggregate stability and water holding capacity), and promoting soil C sequestration. In this study, restoration modes SA and SG decreased soil pH relative to CK. For example, the SA mode had the lowest pH value at the 0–20 cm soil depth. This is presumably due to restoration-induced changes in plant residue decomposition and root processes (Hong et al., 2018). Meanwhile, restoration mode had a significant effect on SOC stocks, and the highest value of SOC stock was observed in the SG mode. This result has two explanations. First, compared with CK and SA, higher plant richness in the SG mode increased plant productivity through niche complementary effects, and consequently, improved plant C inputs into the soil and enhanced SOC accumulation (Chen S. et al., 2018; Chen X. et al., 2019; Li et al., 2019; Jia et al., 2021). Second, SG enhanced soil N and P content more effectively, which played an important role in SOC accumulation by affecting primary productivity and SOC decomposition (Averill and Waring, 2018; Chen et al., 2018a,^13,14^; Wang et al., 2020; Ding et al., 2021). Moreover, we found that ecological restoration significantly increased soil TN, but had no significant effect on TP, in line with a recent meta-analysis (Tian et al., 2021). This could be mainly because, unlike nitrogen, the external source of phosphorus is limited. For instance, diazotrophic microbes can enhance soil N content because of their immense N-fixation ability (Hsu and Buckley, 2009; Xu et al., 2019). Furthermore, SOC mineralization can produce soil nitrogen, hence, a high SOC increases the TN (Tan et al., 2021).

It is generally accepted that MBC, EOC, and HWEOC are the most labile fractions of SOC, which is easily decomposed and mineralized by soil microorganisms (Liang et al., 2021; Xiao et al., 2022). Here, we found that ecological restoration positively affected MBC, EOC, and HWEOC content. Meanwhile, the variation trend of these labile C fractions under ecological restoration was basically similar to that of SOC with a good positive correlation, suggesting that the concentration of these labile C fractions was mainly determined by the plant carbon input. Recently, the ratio of HWEOC to SOC (HWEOC/SOC) was chosen as a chemical index to describe SOC stability (Plante et al., 2011; Hou et al., 2021). The higher HWEOC/SOC, the faster the nutrient cycling rate, which is not conducive to the accumulation of SOC, so the chemical stability is worse and SOC decomposes more easily (Wang et al., 2021). Our result indicated that HWEOC/SOC in the SG mode was higher than in the CK and SA modes, suggesting that SOC in SG easily decomposed and transformed.

### Effects of ecological restoration on the bacterial community and its potential function

Our results revealed that bacterial alpha diversity in SA and SG modes was higher than in CK. Furthermore, bacterial alpha diversity was positively correlated with SOC, TN, TP, HWEOC, and EOC. The evidence suggests that higher labile C and available nutrients in the SA and SG modes can create a more suitable microenvironment for bacterial communities to survive, and thus enhance bacterial alpha diversity. In addition, higher plant richness in the SA and SG modes may provide the bacteria with greater accessibility to a variety of root exudates, which results in more niches to support higher bacterial diversity.

According to the oligotrophic-copiotrophic theory, *Proteobacteria* and *Bacteroidetes* are generally classified as copiotrophic microbes, whereas *Choroflexi* is classified as oligotrophic microbes (Ho et al., 2017; Yao et al., 2017). Our results indicated that modes SA and SG increased the relative abundance of *Proteobacteria* and *Bacteroidetes* but decreased the relative abundance of *Choroflexi*. Correlation analysis indicated that TN, TP, HWEOC, and MBC were positively correlated with *Proteobacteria* and *Bacteroidetes* but negatively correlated with *Choroflexi*, suggesting that copiotrophic taxa gain more advantages in competition due to ecological restoration-induced increasing labile C pools and to nutrient availability. The relative abundance of *Actinobacteria* in SA and SG modes was lower than in CK. Meanwhile, we found a significant positive relationship between pH and *Actinobacteria* under ecological restoration (Supplementary Figure S2). This indicated that the restoration-mediated decrease of pH may decrease their ability to compete with other bacteria taxa (Fu et al., 2022). The negative relationship between *Actinobacteria* and HWOEC and EOC under ecological restoration may be due to some taxa of *Actinobacteria* being oligotrophic groups (Zhong et al., 2019). Additionally, SA and SG increased the relative abundance of *Acidobacteria* relative to CK. Recent studies have found that *Acidobacteria* is a keystone taxon in soil and is involved in the decomposition of soil organic matter (Costa et al., 2020), nitrogen cycling, and plant growth promotion (Eichorst et al., 2018; Kalam et al., 2020). A positive relationship was observed between TN and *Acidobacteria* (Supplementary Figure S2), indicating that the changes in *Acidobacteria* may be tightly linked to soil nitrogen content.

In our study, the Mantel test revealed that soil pH was a major driver of bacterial community composition, which was in line with previous studies on regional studies and large scales (Maestre et al., 2015; Cheng et al., 2020; Hermans et al., 2020). This may be due to the relatively narrow optimal pH for bacterial growth. Many previous studies have demonstrated the important role of soil labile C fractions in shaping soil bacterial communities (Delgado-Baquerizo et al., 2016; Ren et al., 2018; Ramírez et al., 2020). Fundamentally, ecological restoration considerably affected the amount and quality of soil C fractions, which in turn altered microbial community composition (Hu et al., 2022). Our results showed that bacterial community composition was more sensitive to labile C fractions (particularly EOC and HWEOC) than SOC. This suggested that soil labile C fractions could be a critical predictor for bacterial community composition changes in ecological restoration.

Recent evidence has indicated that ecological restoration provides favorable environments for soil carbon functional microbes and stimulates soil C turnover (Guo et al., 2018; Sun and Badgley, 2019; Hu et al., 2022; Li et al., 2022). In our study, the relative abundance of C-fixation genes (*rbcL*, *meh*, *mct*, *ppc*, *IDH1*, and *frdA*) in SA and SG were higher than in CK, suggesting that soil microbes in SA and SG have a strong ability to fix carbon and thus increase the accumulation of SOC. Meanwhile, the relative abundance of *csxA*, *glgX*, *malQ*, and *PYG* genes increased dramatically under SA and SG. This indicated that ecological restoration also improved the microbial decomposition of C sources, and consequently increased CO_2_ production.

### Effects of ecological restoration on C-cycling enzyme activities

Typically, cellulases, *β*-1,4-glucosidase (BG), and *β*-d-cellobiohydrolase (CBH), are related to the degradation of labile C pools, while ligninases, polyphenol oxidase (PPO) and peroxidase (POD) are associated with degradation of recalcitrant C pools (Zhang B. et al., 2021; Zhang S. et al., 2021). Our results showed that ecological restoration had a stronger positive effect on cellulase activity rather than ligninase activity. On the one hand, increasing plant richness under ecological restoration may exhibit stronger niche partition and consequently improve primary productivity as well as soil labile and recalcitrant C pools (Mahaut et al., 2019; Michalet et al., 2021). In this situation, microbes may preferentially invest energy in cellulase production to acquire labile resources over ligninase production since cellulase synthesis requires less energy than ligninase synthesis (Wang et al., 2012; Chen et al., 2018a,b; Chen S. et al., 2018). Our results found positive relationships between labile C fractions and cellulase activity (Supplementary Figure S4), indicating that ecological restoration could enhance cellulase activity *via* increasing labile C substrates. On the other hand, ecological restoration-induced changes in microbial biomass and community composition may also impact enzyme activities (Wu et al., 2021). Positive relationships between cellulase activities, MBC, and SOC (Supplementary Figure S4), indicated that faster microbial degradation and transformation of labile C substrates mediate the accumulation of SOC in SA and SG modes. Moreover, ecological restoration enhanced the relative abundance of copiotrophic microbes (Zeng et al., 2017; Yao et al., 2018; Wang S. et al., 2022; Wang Y. et al., 2022). These microbes had a higher investment in extracellular enzymes to decompose the labile C substrates (Ramin and Allison, 2019). Our results indicated that the cellulase activity was positively correlated to the relative abundance of copiotrophic taxa (*Proteobacteria* and *Bacteroidetes*) (Supplementary Figure S2), which provided evidence that ecological restoration-induced changes in bacterial community composition could affect the response of cellulase activity. Notably, we observed that the ratio of ligninase to cellulase was negatively correlated with SOC content and stocks under ecological restoration (Supplementary Figure S5). This finding indicated that the decreased ratio of ligninase to cellulase under ecological restoration could be benefitical to the accumulation of SOC under ecological restoration, which was consistent with a recent meta-analysis (Wu et al., 2022).

### Effects and mechanisms of ecological restoration on SOC mineralization

Determining the underlying mechanisms controlling SOC mineralization under ecological restoration is challenging since SOC mineralization is regulated by complex factors, including soil physiochemical properties, SOC quality and availability, enzyme activities and soil microbiota. Here, we observed that ecological restoration had a significant effect on the cumulative C mineralization and C mineralization efficiency. The cumulative C mineralization in SA and SG was higher than in CK. The fundamental explanation for the increased C release is that SOC stock was elevated by ecological restoration, which is supported by the substantial positive relationship between cumulative C mineralization and SOC content and stocks. Meanwhile, structural equal modeling revealed that SOC was the main factor driving C mineralization under ecological restoration. In addition to SOC stock, soil N and P content also mediate SOC mineralization by altering microbial activity and community composition (Meyer et al., 2018), which is supported by the positive association between TN and TP and cumulative SOC mineralization.

This study indicated that MBC and HWEOC could better predict the variation in the cumulative C mineralization than SOC. Indeed, higher labile C contents can boost microbial activity and thus stimulate soil C mineralization (Dong et al., 2022). Our SEM showed that MBC had a direct and positive effect on C mineralization. MBC is the C content of live and dead microorganisms, which has a faster turnover and is often used to define soil microbial biomass (Chen C. et al., 2019; Chen X. et al., 2019). When HWEOC is abundant, microbial biomass becomes a major factor limiting C mineralization, thereby playing a critical role in C mineralization (Dong et al., 2022).

Microbial enzymes are “sensors” of microbial function and can provide useful links between microbes and C cycling (Ashraf et al., 2021; Hu et al., 2023). In our study, we observed that BG was significantly and positively associated with cumulative C mineralization, which is in line with previous studies (Zhu et al., 2014; Zhang B. et al., 2021; Zhang S. et al., 2021). SEM results further indicated that C-cycling enzyme activities had a direct and positive effect on C mineralization. This suggested a limitation of enzyme activities on substrate conversion and consumption in degraded grassland soils.

The diversity and composition of the soil microbial community play essential roles in regulating SOC decomposition in terrestrial ecosystems (Qin et al., 2021; Chen et al., 2023). In this study, the Shannon index for bacteria was positively correlated to cumulative C mineralization. In general, soils with higher bacterial diversity may boost the levels of soil microbial functions due to the high functional redundancy of the soil bacterial community (Philippot et al., 2013; Louca et al., 2018; Maron et al., 2018), corroborating the positive correlations between bacterial diversity and enzyme activities, MBC, TN, and TP. Meanwhile, higher bacterial diversity may also sustain plant richness and plant C inputs (van der Heijden et al., 2008; Chen J. et al., 2020; Chen Q. et al., 2020), and finally promote SOC mineralization. Random forest analysis revealed that bacterial community composition played a critical role in controlling SOC mineralization. SEM results showed that bacterial community composition had no significant effect on SOC mineralization but had a significant effect on MBC and C-cycling enzyme activities. This highlighted that bacterial community composition was a crucial underlying factor controlling SOC mineralization *via* mediating microbial production and microbial functionality.

Additionally, soil depth was a key factor that predicted the variation in SOC mineralization. The interpretation was that the SOC pool, labile C content, enzyme activities, and the diversity and activities of soil microorganisms decreased with increasing soil depth, resulting in a decreased SOC mineralization rate (Qin et al., 2021). Overall, our findings suggested that soil physiochemical characteristics, labile C fractions, and the diversity and composition and function of the bacterial community jointly determined the response of SOC mineralization to ecological restoration.

### Effects of ecological restoration on SOC mineralization efficiency

Soil organic carbon mineralization efficiency is crucial in regulating the C cycle and determining the magnitude of soil CO_2_ emissions, thus playing an important role in mitigating climate change. A low SOC mineralization efficiency can result in more C accumulated in the soil, thereby benefiting soil fertility and plant growth. In this study, we found that ecological restoration significantly decreased the SOC mineralization efficiency. Ecological restoration increased large soil aggregates to make inner SOC physically stable and protect it from microbial decomposition. In addition, ecological restoration can change soil physiochemical properties (e.g., pH and texture), which may affect the compositions and activities of soil microbial communities, thereby impacting SOC mineralization efficiency (Dong et al., 2022).

## Conclusion

Ecological restoration had a positive effect on SOC content and stocks, TN, the contents of labile C fractions, cellulase activity, and microbial diversity, whereas decreased soil pH and the ratio of ligninase to cellulase. Soil pH, TN, EOC, and HWOEC were major factors that determining bacterial community composition. Ecological restoration increased the SOC mineralization, but decreased the SOC mineralization efficiency. SOC, MBC and C-cycling enzyme activities had a positive effect on SOC mineralization. Bacterial community composition can regulate SOC mineralization *via* boosting microbial biomass and C-cycling enzyme activities. Our results indicate that shrub with *Salix cupularis* plus grasses had a better SOC accumulation, microbial diversity and functions, which was an optimum mode for restoring alpine degraded grassland.

## Data availability statement

The datasets presented in this study can be found in online repositories. The names of the repository/repositories and accession number(s) can be found at: https://www.ncbi.nlm.nih.gov/, PRJNA915791.

## Author contributions

YH: conceptualization, supervision, funding acquisition, and reviewing and editing. XS and WJL: data collection, data analysis, and writing – original draft. LX, YYZ, WZ, YLZ, and WLL: writing – review and editing. All authors contributed to the article and approved the submitted version.

## Funding

This research was supported by the National Natural Science Foundation of China (41771552) and the Sichuan Science and Technology Program (2020JDRC0074, 2021JDRC0082 and 2022YFS0469).

## Conflict of interest

The authors declare that the research was conducted in the absence of any commercial or financial relationships that could be construed as a potential conflict of interest.

## Publisher’s note

All claims expressed in this article are solely those of the authors and do not necessarily represent those of their affiliated organizations, or those of the publisher, the editors and the reviewers. Any product that may be evaluated in this article, or claim that may be made by its manufacturer, is not guaranteed or endorsed by the publisher.
