# Supplementary material for: Linking between soil properties, bacterial communities, enzyme activities, and soil organic carbon mineralization under ecological restoration in an alpine degraded grassland
Source: Front Microbiol. 2023 Apr 6;14:1131836. doi: 10.3389/fmicb.2023.1131836 (PMC10167489; doi:10.3389/fmicb.2023.1131836)
Supplement: Supplementary file 1 [file Table_1.docx]

**Supplementary materials**

**Supplementary Table S1** Effects of different restoration modes on vegetation characteristics

| Restoration modes | Richness | Coverage % | AGB (g m^-2^) | UGB (g m^-2^) |
| --- | --- | --- | --- | --- |
| CK | 1.0±0.0 d | 4.2±1.9 b | 9.4±10.2 b | 8.5±5.0 c |
| SA | 3.5±1.0 c | 6.6±1.4 b | 32.5±19.2 b | 806.9±341.4 bc |
| SG | 14.0±0.8 a | 81.4±8.9 a | 201.8±39.6 a | 1630.8±749.0 b |

**Supplementary Table S2** *F* values (*P*) for the influences of the restoration mode, soil depth, and restoration mode-soil depth interaction on the soil physical and chemical properties.

| Impact factor | pH | | BD | | SOC | | TN | | TP | | SOC stock | |
| --- | --- | --- | --- | --- | --- | --- | --- | --- | --- | --- | --- | --- |
|  | *F* | *P* | *F* | *P* | *F* | *P* | *F* | *P* | *F* | *P* | *F* | *P* |
| mode | 67.577 | **< 0.001** | 0.659 | 0.53 | 18.071 | **< 0.001** | 91.852 | **< 0.001** | 1.362 | 0.281 | 17.967 | **< 0.001** |
| depth | 6.358 | **0.0213** | 0.019 | 0.893 | 1.113 | 0.305 | 0.026 | 0.874 | 0.052 | 0.822 | 1.034 | 0.323 |
| mode*depth | 3.996 | **0.0366** | 0.296 | 0.748 | 1.113 | 0.350 | 0.504 | 0.612 | 2.615 | 0.101 | 1.065 | 0.366 |

**Supplementary Table S3** *F* values (*P*) for the influences of the restoration mode, soil depth, and restoration mode-soil depth interaction on the soil labile carbon fractions.

| Impact factor | MBC | | EOC | | HWEOC | | HWEOC/SOC | |
| --- | --- | --- | --- | --- | --- | --- | --- | --- |
|  | *F* | *P* | *F* | *P* | *F* | *P* | *F* | *P* |
| mode | 6.935 | **< 0.01** | 60.204 | **< 0.001** | 93.345 | **< 0.001** | 8.429 | **< 0.01** |
| depth | 2.538 | 0.12858 | 4.322 | 0.0522 | 6.197 | **0.023** | 2.008 | 0.1735 |
| mode*depth | 4.262 | **0.03053** | 4.054 | **0.0352** | 1.806 | 0.193 | 0.602 | 0.55824 |

**Supplementary Table S4** *F* values (*P*) for the influences of the restoration mode, soil depth, and restoration mode-soil depth interaction on the soil C-cycling enzymes and the ratio of ligninase to cellulase.

| Impact factor | BG | | CBH | | POD | | PPO | | LC | |
| --- | --- | --- | --- | --- | --- | --- | --- | --- | --- | --- |
|  | *F* | *P* | *F* | *P* | *F* | *P* | *F* | *P* | *F* | *P* |
| mode | 10.369 | **< 0.01** | 4.877 | **0.0203** | 2.443 | 0.115 | 0.237 | 0.792 | 8.387 | **<0.01** |
| depth | 1.745 | 0.20308 | 0.029 | 0.8668 | 0.215 | 0.648 | 0.238 | 0.632 | 0.405 | 0.53266 |
| mode*depth | 0.475 | 0.62935 | 1.479 | 0.2543 | 0.612 | 0.553 | 0.122 | 0.886 | 0.043 | 0.95822 |

**Supplementary Table S5** *F* values (*P*) for the influences of the restoration mode, soil depth, and restoration mode-soil depth interaction on the soil C-cycling enzymes and the ratio of ligninase to cellulase.

| Impact factor | Cmin | | CME | |
| --- | --- | --- | --- | --- |
|  | *F* | *P* | *F* | *P* |
| mode | 59.22 | **< 0.001** | 1.039 | 0.374155 |
| depth | 166.07 | **< 0.001** | 15.783 | **< 0.001** |
| mode*depth | 20.06 | **< 0.001** | 1.95 | 0.171213 |
