# Supplementary material for: Linking between soil properties, bacterial communities, enzyme activities, and soil organic carbon mineralization under ecological restoration in an alpine degraded grassland
Source: Front Microbiol. 2023 Apr 6;14:1131836. doi: 10.3389/fmicb.2023.1131836 (PMC10167489; doi:10.3389/fmicb.2023.1131836)
Supplement: Supplementary file 2 [file Data_Sheet_1.docx]

**Supplementary Figure**

**Supplementary Figure S1** Location of the study area and image of ecological restoration modes. Note: CK, extremely degraded grassland; SA, planting shrub with *Salix cupularis* alone; SG, planting shrub with *Salix cupularis* plus grasses.

**Supplementary Figure S2** Relationships between the diversity and dominant phyla of bacterial community and soil physicochemical properties, labile C fractions, C-cycling enzymes, and SOC mineralization.

**Supplementary Figure S3** Redundancy analysis (RDA) of bacterial community changes with soil properties.

**Supplementary Figure S4** Relationships between C-cycling enzyme activities and soil properties.

**Supplementary Figure S5** Relationships between the ratio of ligninase to cellulase and SOC content and stocks under ecological restoration.
