# Supplementary figures and images for: Linking between soil properties, bacterial communities, enzyme activities, and soil organic carbon mineralization under ecological restoration in an alpine degraded grassland
Source: Front Microbiol. 2023 Apr 6;14:1131836. doi: 10.3389/fmicb.2023.1131836 (PMC10167489; doi:10.3389/fmicb.2023.1131836)

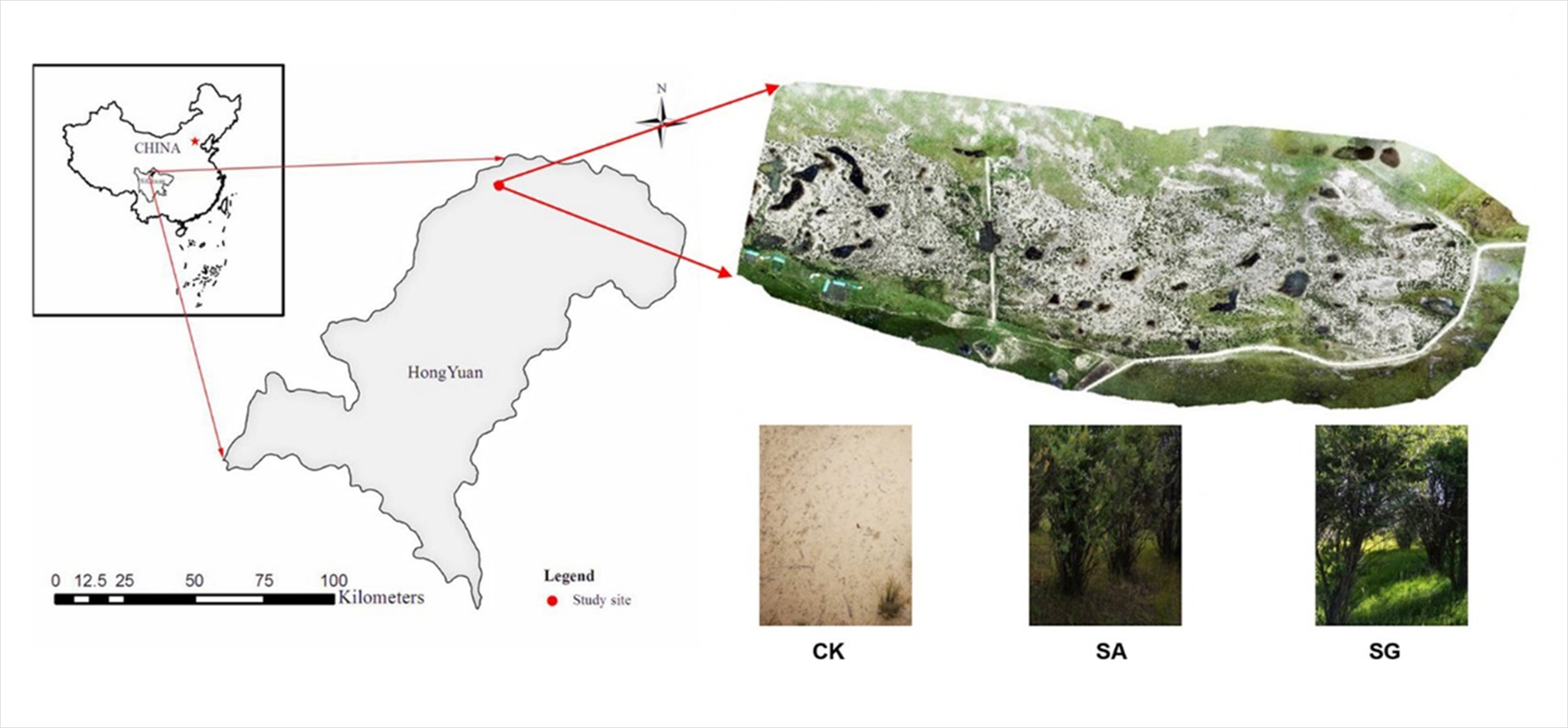

Supplement: Supplementary file 3 [file Image_1.tif]

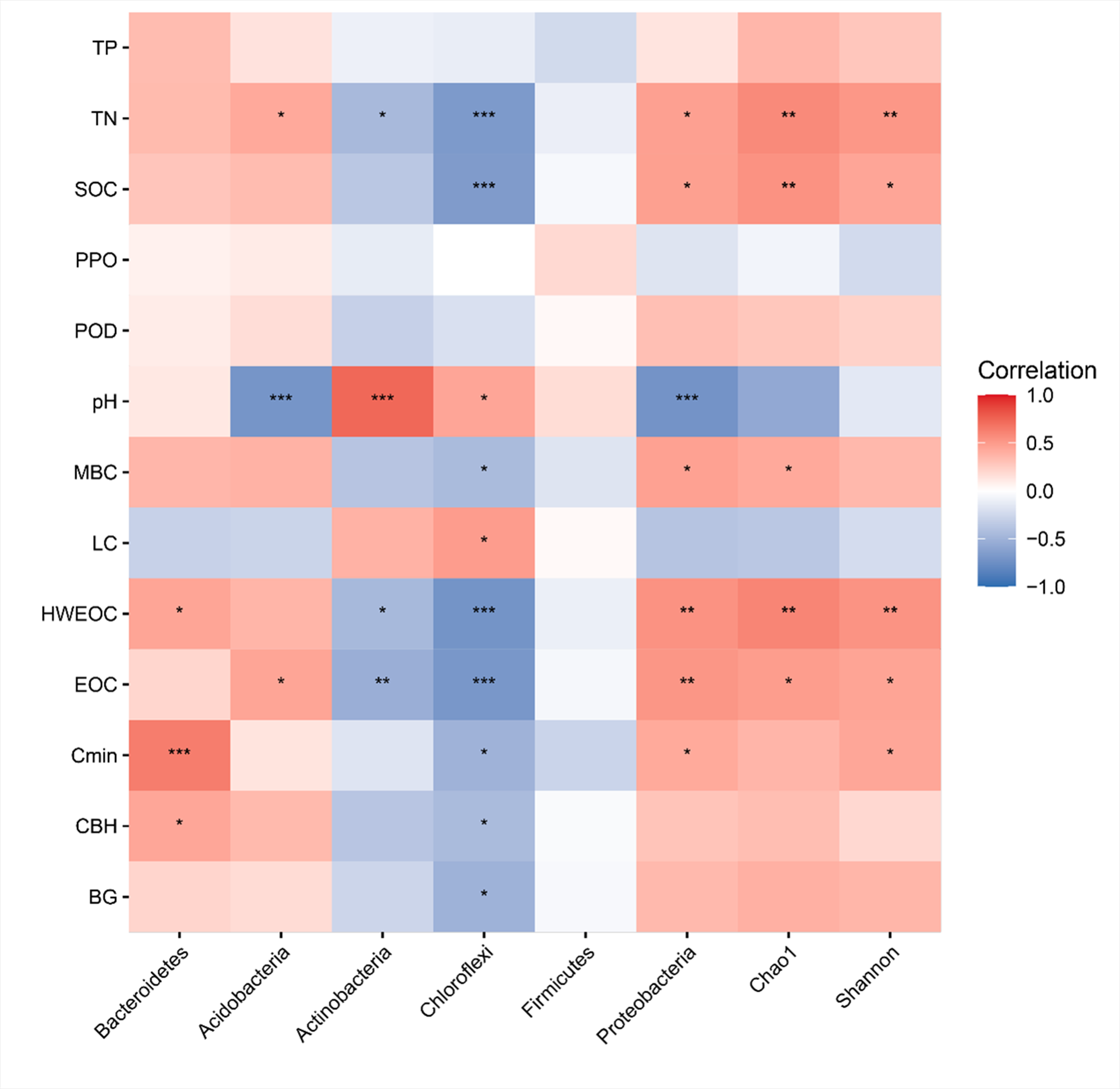

Supplement: Supplementary file 4 [file Image_2.tif]

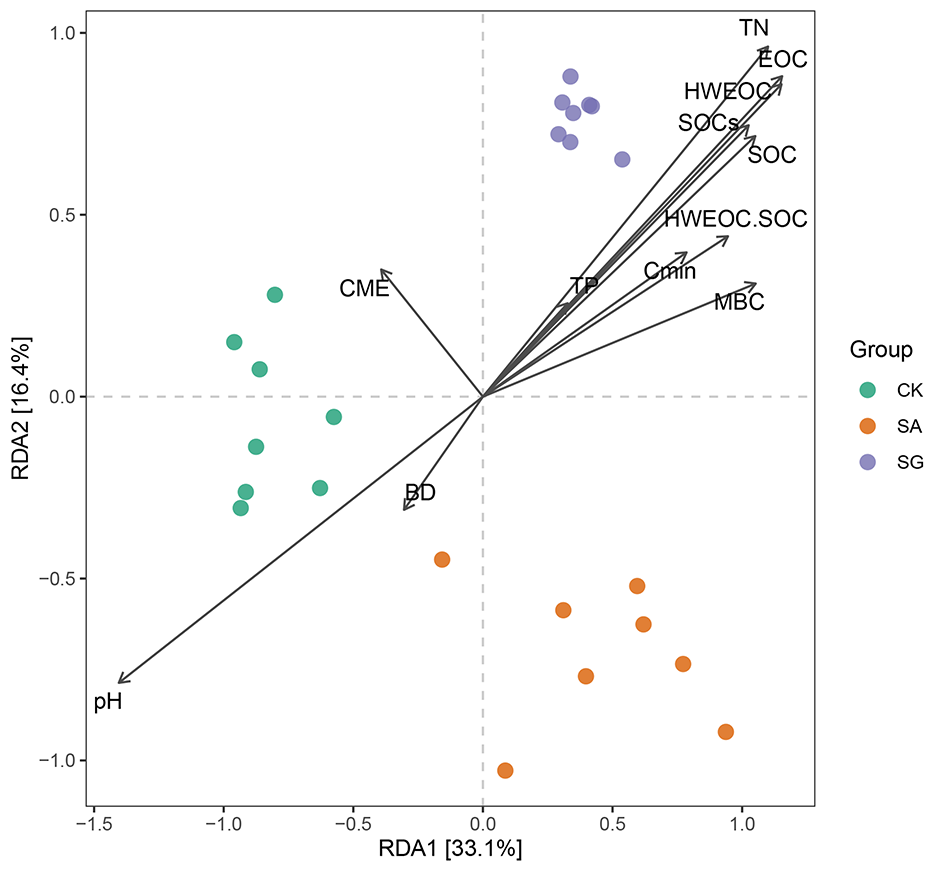

Supplement: Supplementary file 5 [file Image_3.tif]

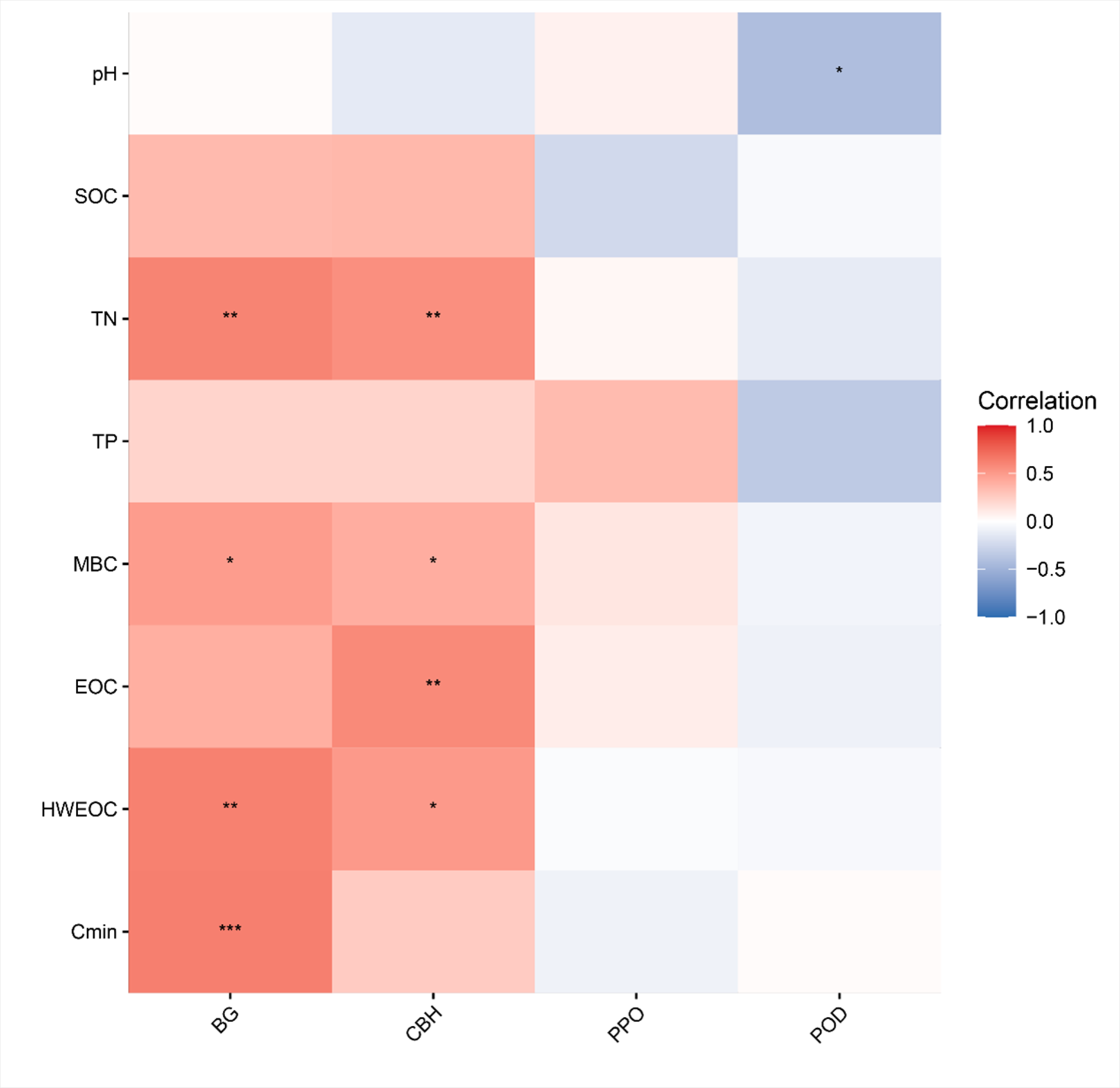

Supplement: Supplementary file 6 [file Image_4.tif]

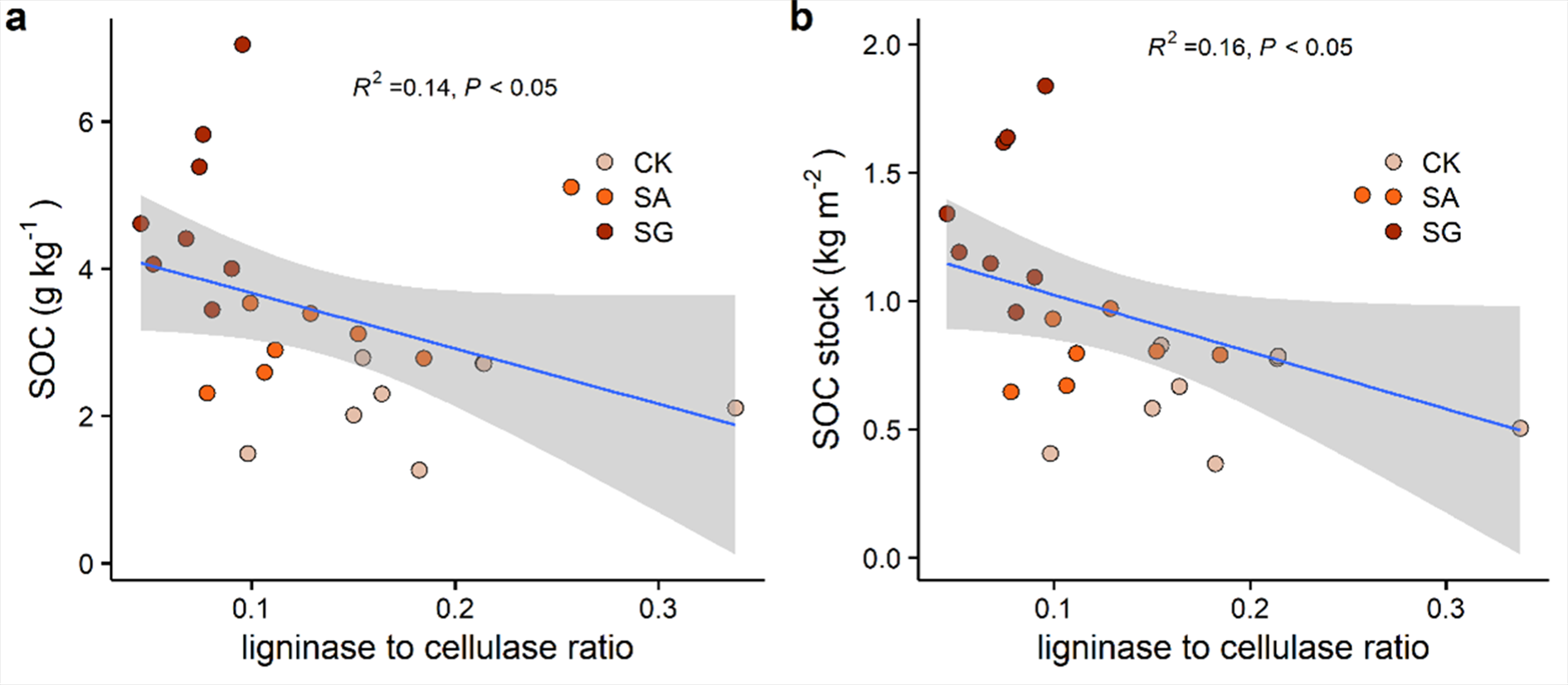

Supplement: Supplementary file 7 [file Image_5.tif]
